# Transmission Dynamics of *Borrelia turicatae* from the Arthropod Vector

**DOI:** 10.1371/journal.pntd.0002767

**Published:** 2014-04-03

**Authors:** William K. Boyle, Hannah K. Wilder, Amanda M. Lawrence, Job E. Lopez

**Affiliations:** 1 Department of Biological Sciences, Mississippi State University, Starkville, Mississippi, United States of America; 2 Institute for Imaging and Analytical Technologies, Mississippi State University, Starkville, Mississippi, United States of America; University of California San Diego School of Medicine, United States of America

## Abstract

**Background:**

With the global distribution, morbidity, and mortality associated with tick and louse-borne relapsing fever spirochetes, it is important to understand the dynamics of vector colonization by the bacteria and transmission to the host. Tick-borne relapsing fever spirochetes are blood-borne pathogens transmitted through the saliva of soft ticks, yet little is known about the transmission capability of these pathogens during the relatively short bloodmeal. This study was therefore initiated to understand the transmission dynamics of the relapsing fever spirochete *Borrelia turicatae* from the vector *Ornithodoros turicata*, and the subsequent dissemination of the bacteria upon entry into murine blood.

**Methodology/Principal Findings:**

To determine the minimum number of ticks required to transmit spirochetes, one to three infected *O. turicata* were allowed to feed to repletion on individual mice. Murine infection and dissemination of the spirochetes was evaluated by dark field microscopy of blood, quantitative PCR, and immunoblotting against *B. turicatae* protein lysates and a recombinant antigen, the *Borrelia* immunogenic protein A. Transmission frequencies were also determined by interrupting the bloodmeal 15 seconds after tick attachment. Scanning electron microscopy (SEM) was performed on infected salivary glands to detect spirochetes within acini lumen and excretory ducts. Furthermore, spirochete colonization and dissemination from the bite site was investigated by feeding infected *O. turicata* on the ears of mice, removing the attachment site after engorment, and evaluating murine infection.

**Conclusion/Significance:**

Our findings demonstrated that three ticks provided a sufficient infectious dose to infect nearly all animals, and *B. turicatae* was transmitted within seconds of tick attachment. Spirochetes were also detected in acini lumen of salivary glands by SEM. Upon host entry, *B. turicatae* did not require colonization of the bite site to establish murine infection. These results suggest that once *B. turicatae* colonizes the salivary glands the spirochetes are preadapted for rapid entry into the mammal.

## Author Summary

Relapsing fever spirochetes cause recurrent febrile episodes, rigors, nausea, vomiting, malaise, and pregnancy complications, and are a leading cause of hospital admissions in regions of Africa. Routes of pathogen transmission include crushed human body lice and feces, or through bites by *Ornithodoros* spp. of ticks. The life cycle of *Ornithodoros turicatae*, the vector of *Borrelia turicatae*, includes over six nymphal stages, upwards of a ten year life span, and a bloodmeal that is completed within an hour. We investigated *B. turicatae* transmission from the tick vector and assessed the rapidity of spirochete entry into the mammal and dissemination in the blood. Salivary glands from infected ticks were also evaluated to visualize *B. turicatae* within the tissues to determine spirochete localization. We conclude that given the transmission dynamics of *B. turicatae*, it may be important to target conserved surface proteins that relapsing fever spirochetes produce in the salivary glands in order to develop preventative measures against the pathogens.

## Introduction

Vector competency for a microbial agent is defined as the acquisition, maintenance, and subsequent transmission of the pathogen, and is dependent on host seeking behavior, duration of attachment, transstadial passage, and transovarial transmission [1]. Pathogens must attain sufficient densities within the host to promote uptake by the vector then quickly adapt to a new environment, given the physiological and immunological differences between mammal and vector. Transstadial and transovarial passage is essential with pathogens utilizing one or both routes [1], and subsequent transmission is dependent on the feeding behavior of the vector and the microbes ability to efficiently infect the host. Understanding the interplay between mammalian host, pathogen, and vector is essential toward disease control.

Two families of ticks, Argasidae (soft ticks) and Ixodidae (hard ticks), have evolved different feeding behaviors. Argasid ticks generally engorge within an hour while ixodids take several days. Most vector competency and transmission studies of tick-borne pathogens have focused on microbes transmitted by ixodid ticks [2]–[8]. Less is known concerning the transmission process of soft tick species within the genus *Ornithodoros*, which display a different feeding behavior, yet also transmit pathogens of public health relevance, including tick-borne relapsing fever spirochetes. Variations in the feeding behaviors between argasid and ixodid ticks may account for the observed differences in transmission between their respective pathogens. For instance, optimal transmission of *Borrelia burgdorferi* and *Anaplasma phagocytophilum* occurs after 48 hours, and the five to seven day bloodmeal of *Ixodes* spp. ensures transmission and the continued life cycle of the pathogens [3], [6], [9], [10]. *Borrelia turicatae*, a species of tick-borne relapsing fever spirochete, is transmitted by *Ornithodoros turicata*, which depending on the developmental stage completes the bloodmeal within 5–60 minutes [11]. The feeding behavior of the *O. turicata* suggests that the adaptation of *B. turicatae* to the vector is much different than pathogens transmitted by ixodid ticks.

The kinetics of *B. turicatae* entry into the host are largely unknown. Using a Swiss Webster animal model, we characterized *B. turicatae* transmission from the arthropod vector. The number of infected third stage nymphs required to successfully transmit spirochetes was determined. We also compared murine infection when ticks fed to repletion or were interrupted within 15 seconds of attachment. Given our results, scanning electron microscopy (SEM) was performed to localize *B. turicatae* within tick salivary glands, and *B. turicatae* colonization and dissemination from the bite site was also evaluated. Our findings demonstrate that three ticks provide a sufficient infectious dose to infect 80–100% of mice, and that transmission and dissemination of *B. turicatae* are rapid events.

## Methods

### Ethical statement

Animal work and husbandry was conducted in adherence to the United States Public Health Service Policy on Humane Care and Use of Laboratory Animals and the Guide for the Care and Use of Laboratory Animals. Animal husbandry was provided under the Association for Assessment and Accreditation of Laboratory Animal Care and Office of Laboratory Animal Welfare assured program at Mississippi State University. The studies were approved by the Mississippi State University Institutional Animal Care and Use Committee (IACUC protocol #11-091).

### Tick colony

*O. turicata* ticks used in this study originated from an uninfected colony maintained at the Rocky Mountain Laboratories, NIAID, NIH, and subsequently Mississippi State University. All ticks were housed at 25°C and 85% relative humidity [12]. To obtain an infected cohort of ticks, two 4 week old Swiss-Webster mice (Harlan Laboratories Inc., Tampa, FL, USA) were needle inoculated with *B. turicatae* 91E135, and 75 uninfected second stage nymphal ticks were fed on each animal when spirochetes were detected in the blood (1×10^6^ spirochetes/ml). After molting, a 3–4 week process, ticks were subsequently fed to evaluate *B. turicatae* transmission. In all experiments, mice were sedated with 25 mg/ml Ketamine and 7.6 mg/ml Xylazine at 0.05 ml/25 g body weight during the bloodmeal.

### Transmission studies

Interrupted feedings and full bloodmeals were performed using 1–3 infected third stage nymphal ticks. Five to 12 mice were fed upon by one to three ticks, and after attachment the animals were individually housed. Ticks that fed to repletion were allowed to attach simultaneously, and the time required for engorgement was recorded. To evaluate rapid transmission, an individual tick was placed on the shaved abdomen of an animal and a timer was started once the tick inserted their mouth parts, as determined by the inability to move the tick with a paintbrush. After 15 sec the tick was removed. When two or three ticks were fed, they were allowed to individually attach for 15 sec, removed, and a subsequent tick was placed on the animal. The total time a given tick was on an animal was also recorded.

### Spirochete detection

Dark field microscopy, quantitative PCR (qPCR), and seroconversion were used to determine infection frequencies after tick bite. For 10 consecutive days after tick bite, a drop of blood (approximately 2 µl) was collected by tail nick onto a slide and examined using a Zeiss Axiovert (Thornwood, NY, USA) dark field microscope. Thirty fields were scanned for spirochetes. An additional 2.5 µl of blood was collected from each animal by tail nick and pipetted into 47.5 µl of Lysis-Stabilization Buffer (Agilent, Santa Clara, CA, USA), and the qPCR was performed as previously described with minor modifications [13]. Primers and probe were designed to *B. turicatae flaB* (forward primer: CCAGCATCATTAGCTGGATCAC, reverse primer: GTTGTGCACCTTCCTGAGC, and probe: YAK–TGCAGGTGAAGGTGCGCAGGTT–BBQ). *B. turicatae* cultured in mBSK medium [14], [15] was used to generate a standard curve from 1×10^4^ to 1×10^8^ spirochetes/ml. qPCR assays were performed using the ABI 96-well Step-One Plus Instrument (Life Technologies, Foster City, USA) in triplicate with 20 µl reactions containing 2× Brilliant qPCR Master Mix (Agilent Technologies, Waldbroon, Germany).

To detect spirochetes in the salivary glands from ticks that fed on mice that failed to become infected, immunofluorescent assays (IFA) were performed as previously described using chicken serum generated against *B. turicatae* recombinant flagellin (rFlaB) [16]. The cuticle and midgut were removed and the inner cavity of the *O. turicata* was rinsed with PBS - 5 mM MgCl_2_. Salivary glands were removed, crushed, adhered to a cover slip by heat, and fixed in acetone for 30 min. The chicken anti-rFlaB was diluted 1∶20 in PBS - 0.75% BSA, and the secondary antibody was an Alexa Fluor 568 Goat Anti-Chicken IgG (Life Technologies) diluted 1∶200. Spirochetes were visualized using a Zeiss Axioskop 2 Plus (Carl Zeiss Microscopy, Munich, Germany).

### SDS-PAGE and serological analysis

Four weeks after tick feedings, blood was collected from mice and immunoblotting was performed as previously described [17]. Protein lysates from 1×10^7^ spirochetes and 1 µg of recombinant *Borrelia* immunogenic protein A (rBipA) were analyzed using Mini-PROTEAN TGX precast gels (Bio-RAD, Hercules, CA). Gels were transferred to Immobilon PVDF membrane (Millipore, Billerica, MA, USA). Immunoblots were probed for one hour with mouse sera diluted at 1∶500 and the secondary molecule HRP-rec-Protein A (Life Technologies) was applied at a 1∶4,000 dilution for one hour. Serological reactivity was determined by chemiluminescence using the Amersham ECL Western Blotting Detection Reagents (GE Healthcare Bio-Sciences Corp., Piscataway, NJ).

### Scanning Electron Microscopy (SEM)

To perform SEM on intact salivary glands within the inner cavity of *O. turicata*, the cuticle and midgut were removed, discarded, and the tick was washed with PBS, and placed in Karnovsky's fixative. Individual salivary glands were also excised and placed in Karnovsky's fixative. Samples were treated with 2% Osmium tetraoxide for 2 hours then dehydrated by immersion in increasing concentrations of ethyl alcohol, with the final concentration of 100% ethyl alcohol. Cryofracturing was performed on excised salivary glands in liquid nitrogen. Samples were dried using a E300 Critical Point Dryer (Polaron Equipment. Watford, United Kingdom) and viewed under a JEOL JSM-6500F Field Emission scanning electron microscope (JEOL USA, Inc., Peabody, MA, USA).

### Detection of spirochetes in tissue biopsies

To evaluate dissemination within murine blood, three infected *O. turicata* were allowed to feed to repletion on the ear pinna of sedated Swiss Webster mice, as stated above. Following detachment the ears were rubbed with 7.5% povidone-iodine and rinsed with 70% ethanol. Bite sites were removed using a sterile 2 mm tissue biopsy punch immediately after detachment. To assess colonization of the bite site, tissue biopsies were placed into 5 ml mBSK medium [14], [15], incubated at 35°C for two weeks, and 10 µl of medium was analyzed by dark field microscopy Zeiss Axiovert (Thornwood, NY, USA). Spirochete dissemination in the blood was evaluated by dark field microscopy for 10 consecutive days after tick feeding and immunoblotting using serum samples collected four weeks after feeding ticks as described above.

### Statistics

Data analysis of transmission frequencies was performed using Microsoft Excel (Microsoft, Redmond, WA, USA), and comparisons of spirochete densities within the blood were performed with the Statistical Package for Social Sciences (SPSS) software (IBM Corp., Armonk, NY). Confidence intervals for transmission frequencies were constructed with binomial proportions to compare interrupted feeding and full blood meal treatments. To compare *B. turicatae* blood densities between groups of mice in which ticks were allowed to fully feed or were interrupted 15 sec after attachment, bacterial densities were converted to spirochetes per ml of blood from qPCR C_t_ values as previously described [13]. Confidence intervals were calculated when spirochetes were detected in three or more mice, when murine sample size was sufficient.

## Results

### *B. turicatae* transmission to Swiss Webster mice

**Figure 1:**
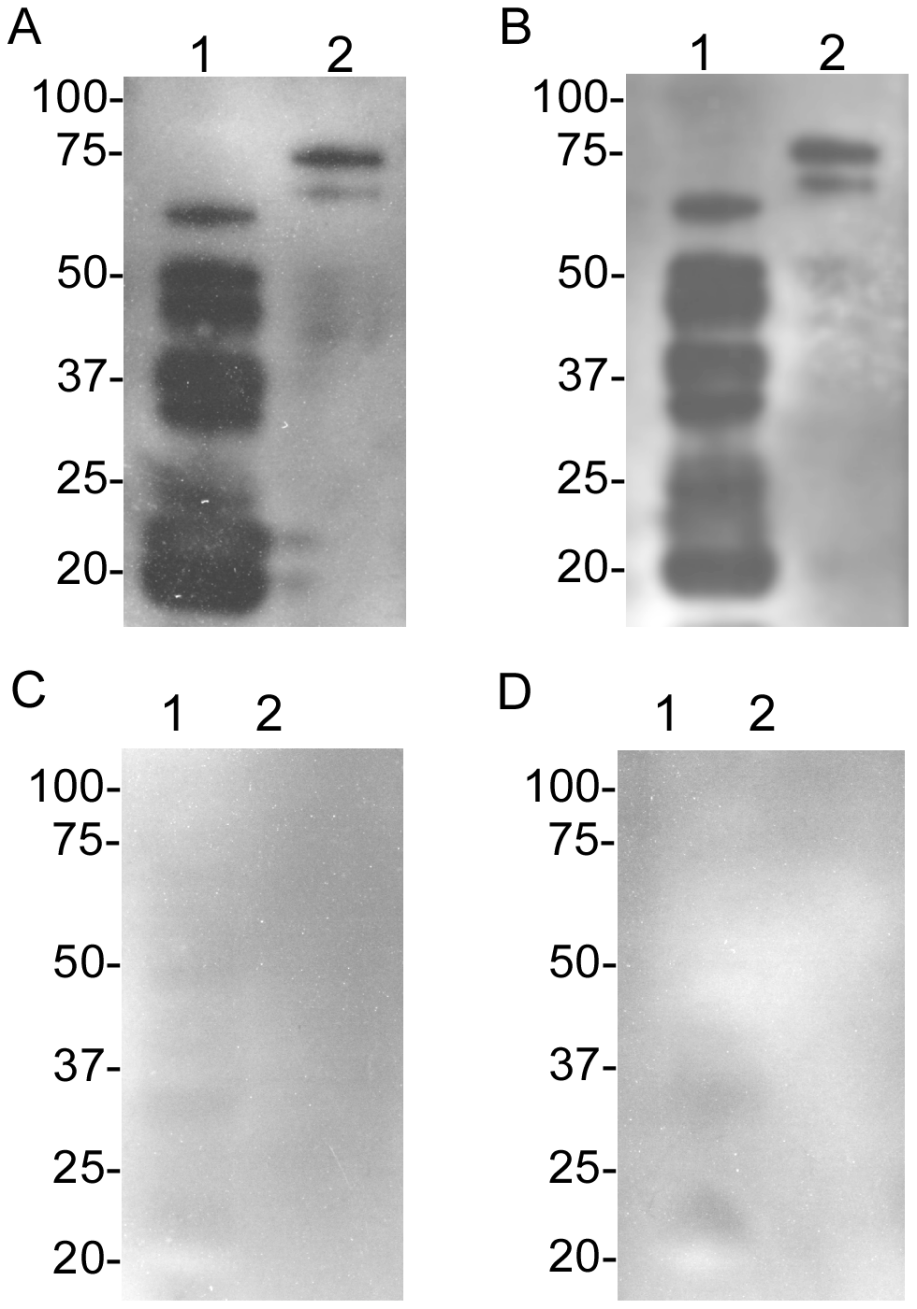
Immunoblots from four mice demonstrating seroconversion in animals when spirochetes were detected by microscopy or qPCR after ticks fed to repletion (A), or were interrupted after attaching for 15 seconds (B). Animals that were fed on by infected ticks yet spirochetes were undetectable in murine blood (C and D). *B. turicatae* protein lysates and rBipA were electrophoresed in lane 1 and 2, respectively. Serum samples were collected from animals four weeks after tick feeding. Molecular masses (kilodaltons) are indicated to the left of each immunoblot.

**Figure 2:**
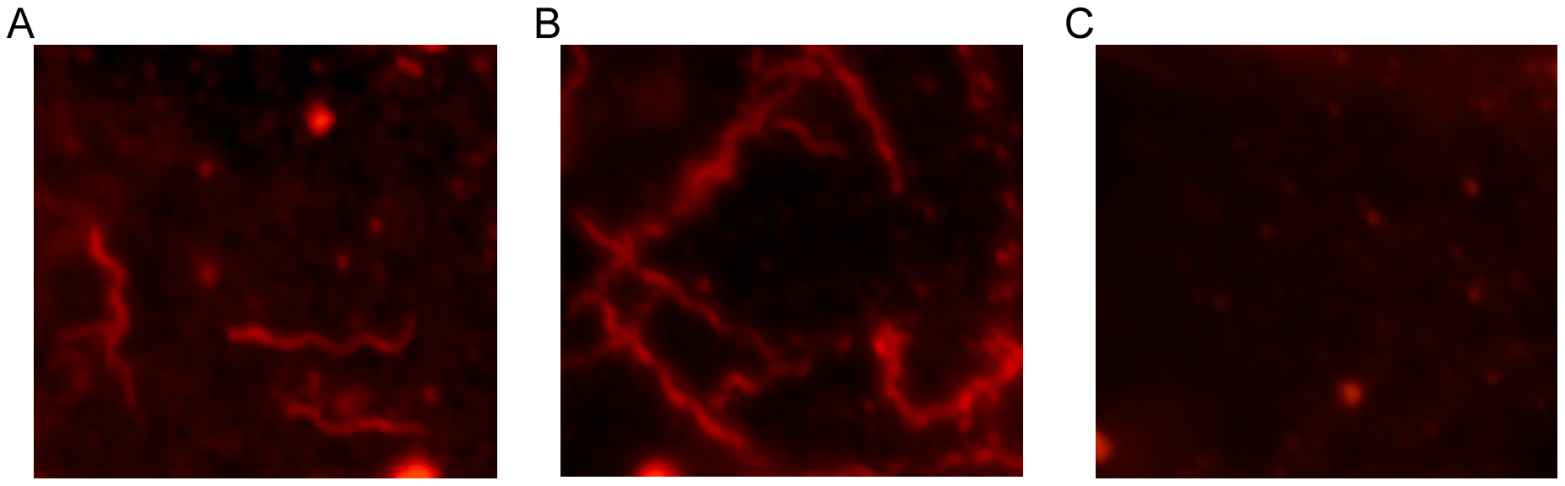
Evaluation of salivary gland colonization by *B. turicatae*. IFA of salivary glands considered infected (A), *in vitro* cultivated spirochetes as a positive control (B), and uninfected tick salivary glands (C). These findings from a single tick are representative of the remaining infected ticks.

The lack of available information regarding transmission efficiencies by *O. turicata* initiated a comparative study feeding one to three third stage nymphal ticks on a given mouse. All ticks engorged within 10–30 minutes. Comparing microscopy, qPCR, and serological responses indicated that immunoblotting and qPCR results were similar and more consistent in assessing murine infection than dark-field microscopy (Table 1). For example, when spirochetes were detected in the blood by microscopy or qPCR, mice seroconverted to *B. turicatae* protein lysates and rBipA (Figure 1 A and B), an antigen for relapsing fever spirochetes [18], [19]. Animals in which spirochetes were not detected in the blood by qPCR failed to seroconvert (Figure 1 C and D). Serological responses from the four mice (Figure 1 A–D) were representative of remaining animals that were fed upon by infected ticks. Murine infection rates after one to three *O. turicata* fed indicated that three ticks provided an infectious dose to a minimum of 80% of mice (Table 1). Also, IFA enabled *B. turicatae* visualization in the salivary glands from 9 of 10 ticks that were individually fed on mice (Figure 2 A–C), suggesting that while most ticks were colonized with spirochetes, they failed to deliver a sufficient infectious dose.

**Table 1:** Comparisons of *B. turicatae* transmission frequencies by dark field microscopy, qPCR, and immunoblotting after an uninterrupted and interrupted bloodmeal when one to three ticks attached.

No. ticks	Uninterrupted bloodmeal			Interrupted bloodmeal		
	Microscopy	qPCR	Immunoblotting	Microscopy	qPCR	Immunoblotting
1	0/5	1/5	1/5	1/5	1/5	1/5
2	1/5	1/5	1/5	1/5	1/5	1/5
3	6/10	8/10	8/10	5/12	7/12	7/12

**Figure 3:**
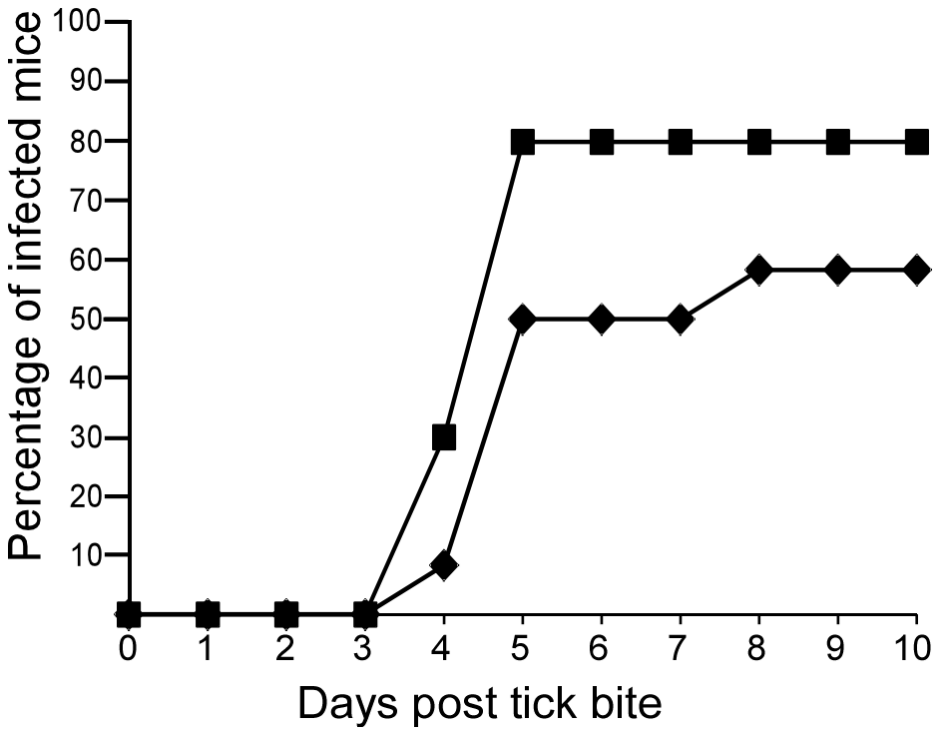
Cumulative frequency of infection detected in mice after transmission. Three infected ticks were fed on mice (n = 22) and spirochetemia was detected by microscopy and qPCR. Successful transmission frequencies after full repletion (squares) and interrupted feeding (diamonds) are separated for comparison.

Evaluating the timing of transmission was performed using *O. turicata* nymphs that originated from the same cohort as the ticks that fed to repletion. Detecting spirochetes after allowing ticks to attach for 15 seconds indicated that transmission was a rapid event (Table 1), and the total time an individual tick was in contact with an animal was less than 40 seconds. Interestingly, transmission frequencies were identical when one or two ticks fed to repletion or were removed 15 seconds after attachment (Table 1). When three ticks were fed on a given animal, differences in infection rates after interrupted and uninterrupted bloodmeals were not statistically significant (Table 1). Also, detection of infection in most mice was determined by the fifth day when three ticks fed to repletion or were interrupted during the bloodmeal (Figure 3).

### Quantification of spirochete densities in the blood after tick bite

**Figure 4:**
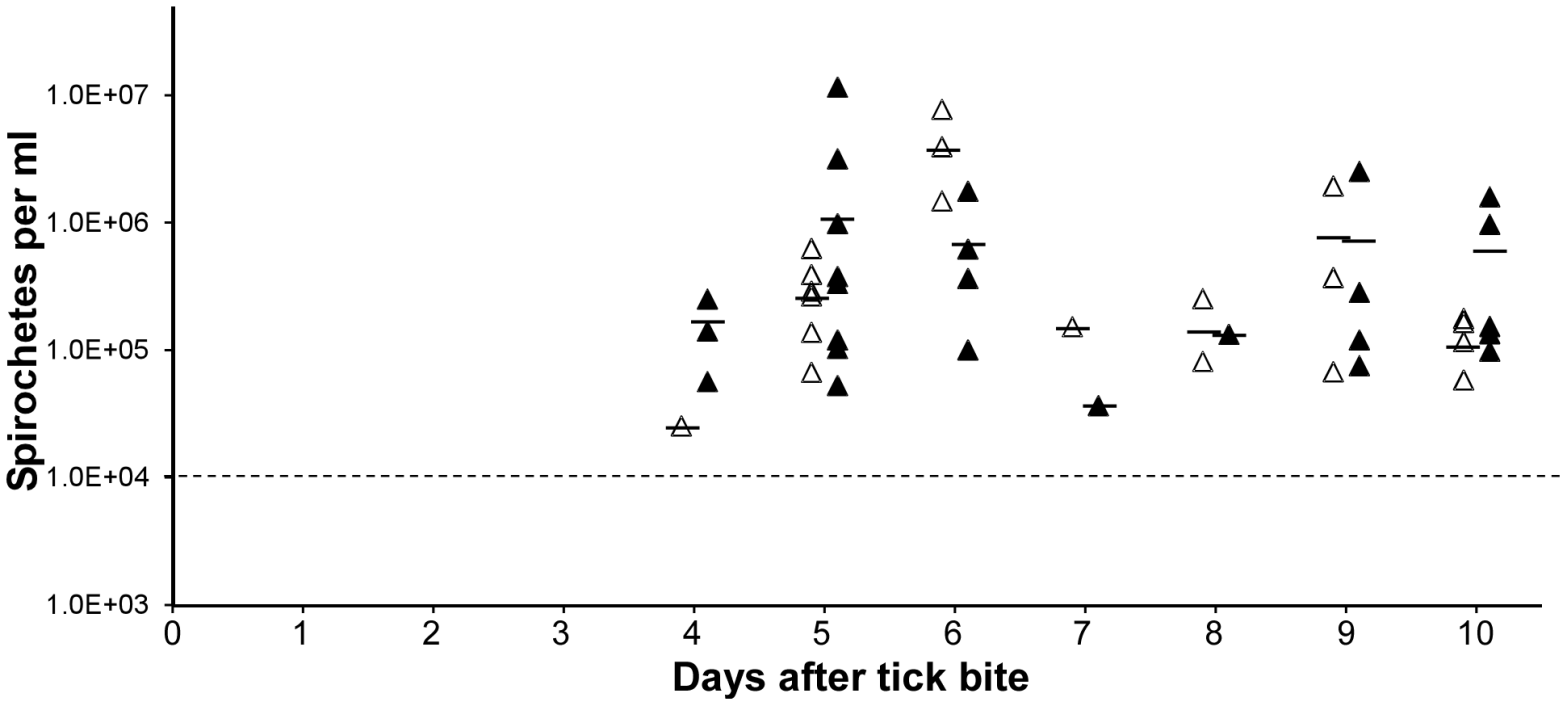
Spirochete densities in murine blood after allowing three infected ticks to engorge (black triangle), or interrupting the bloodmeal after 15 seconds (white triangle). Each triangle represents spirochete densities in a given animal. Solid horizontal lines are the average number of spirochetes per ml of blood among the spirochetemic mice on a given day. The limit of detection is indicated by the horizontal dotted line.

When three ticks engorged or were removed shortly after attachment, the density of the spirochetes within the blood were similar over a 10 day period as determined by qPCR (Figure 4). Spirochetes were detected on the fourth day after tick bite and *B. turicatae* quantities in the blood were comparable regardless whether ticks fed to repletion or were interrupted within 15 seconds of attachment. The progression of infection was cyclic, with *B. turicatae* relapsing in the blood beginning on the seventh day after tick bite, and by the ninth day all animals were once again spirochetemic. Also, spirochete kinetics in the blood when one or two ticks fed on an animal were identical (data not shown). These results suggest that the complete infectious dose delivered by *O. turicata* occurs within 15 seconds of attachment.

### Spirochete colonization of salivary gland acini

**Figure 5:**
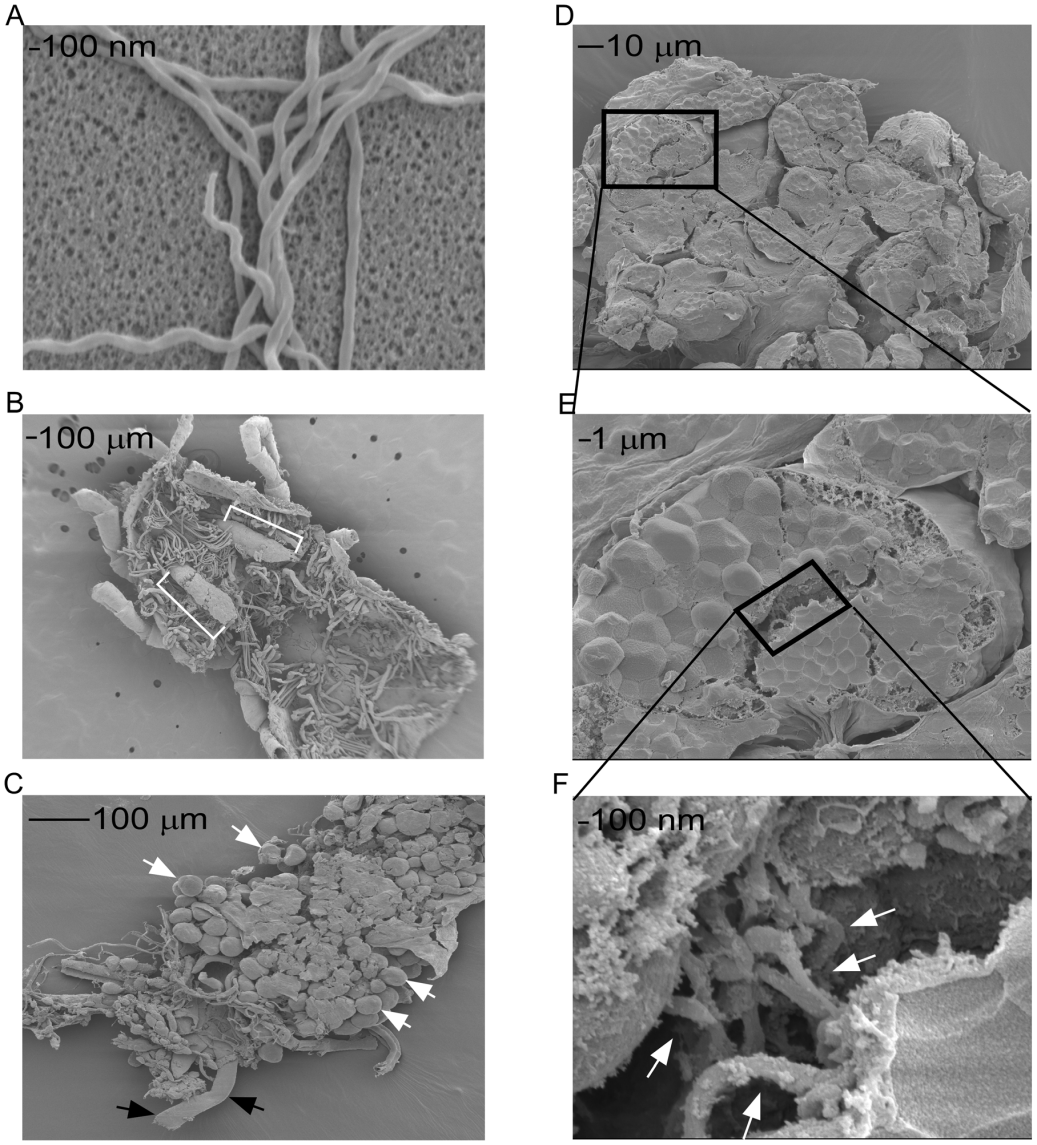
SEM of infected *in vitro* grown *B. turicatae* and salivary glands from infected *O. turicata* (A–F). Images of *in vitro* cultured spirochetes (A) were used as reference for spirochete size and shape. A dorsal view of a tick in which the cuticle and midgut were removed, where white ([ ]) indicate the location of the salivary glands (B). An intact salivary gland was removed and the white and black (→) indicates an individual acinus and excretory duct, respectively (C). A cluster of cryofractured acini (D) and a magnified single acinus (E). The lumen of an ancinus was analyzed for the presence of spirochetes (F), as represented by white (→). The black boxes represent progressively magnified (D–F). The scale in nm and µm are represented by black bars.

Salivary gland acini and excretory ducts from unfed ticks were evaluated for the presence of spirochetes by SEM because colonization of these sites would enable rapid transmission. *B. turicatae* grown *in vitro* provided a reference for spirochete size and morphology (Figure 5 A). Intact salivary glands visualized by SEM indicated the presence excretory ducts and acini (Figure 5 B and C). Analyzing cryofractured salivary glands identified distinguishable spirochetes in a portion of acini lumen (Figure 5 D–F), while bacteria were undetected within cryofractured excretory ducts (data not shown). We also failed to detect spirochete-like organisms in salivary glands of uninfected ticks (data not shown), indicating the identified bacteria were *B. turicatae*.

### *B. turicatae* dissemination from the bite site

*B. turicatae* colonization of the bite site was evaluated after a bloodmeal. Feeding three third stage nymphs to repletion on the ears of five mice and removing the bite site immediately after detachment (Figure S1) indicated that spirochetes disseminated from the bite site during tick feeding (Table 2). All mice became infected on the fourth day after tick bite as determined by dark field microscopy and subsequently by immunoblotting using serum samples collected from the animals four weeks after tick bite (data not shown). Similar results were observed in animals in which the bite site was left intact (Table 2). *B. turicatae* was also cultivated from the ear biopsies, indicating that spirochetes remained viable at the bite site after the bloodmeal. Collectively these results suggest that populations of *B. turicatae* colonize the bite site while others disseminate in the blood and are sufficient to establish an infection in mice.

**Table 2:** Comparing *B. turicatae* infection frequencies in mice when the bite site was removed or left intact after feeding three ticks.

No. ticks	0 hr^A^	Control animals
3	100% (5/5)	100% (5/5)

A bite site was removed immediately after detachment.

## Discussion

Previous transmission studies of *B. turicatae* were conducted over 70 years ago using five to 270 ticks that fed on animals, including mice and nonhuman primates, and a single *O. turicata* larvae was found to deliver a sufficient infectious dose to man [11], [20], [21]. The studies were often performed using field collected *O. turicata* and the numbers of infected ticks within a cohort was unclear [11], [20], [21]. Additionally, transmission was evaluated by visualizing spirochetes in the blood, but densities of *B. turicatae* were not quantified. In this present report, successful transmission from one to three ticks was determined by comparing dark field microscopy, molecular detection with qPCR, and serological responses. We found that qPCR was more reliable at detecting *B. turicatae* in murine blood than dark field microscopy. Failure to seroconvert suggested that infections were not occurring in mice that were negative by qPCR and dark field microscopy.

Characterizing transmission of *B. turicatae* in mice identified interesting differences compared to the *Ornithodoros hermsi-Borrelia hermsii* model of tick-borne relapsing fever spirochetes. In our study, uninfected second stage *O. turicata* nymphs initially engorged when mice were infected with approximately 1,000 spirochetes/µl of blood. During the subsequent bloodmeal, three ticks were required to successfully infect 80–100% of Swiss Webster mice with *B. turicatae*, while one and two ticks provided an infectious dose in 20% of the animals (Table 1 and 2). When the initial acquisition bloodmeal of second stage nymphal *O. hermsi* occurred with nearly 300 spirochetes/µl in murine blood, subsequent successful transmission frequencies from single ticks to Swiss Webster mice were 96% [22]. Furthermore, upon transmission, the average densities of *B. turicatae* in mice were nearly a log lower than observed with *B. hermsii*, which attain over 1×10^7^ spirochetes/ml [23]. *B. hermsii* is naturally maintained in mice, squirrels, and chipmunks [24], and successful transmission in rodents ensures the continued life-cycle of the spirochetes. The maintenance of *B. turicatae* in nature is less understood. The ecological niche of the tick vector overlaps that of feral swine (unpublished observations), wild canids, bats, and the gopher tortoise [25]–[28], suggesting *B. turicatae* may be adapted to infect these vertebrates more efficiently than mice.

The rapidity of *B. turicatae* transmission by tick bite was initially suggested by Dr. Gordon Davis. He reported that male *O. turicata* fed to repletion within 6 to 23 minutes, and transiently mentioned that transmission could occur within a minute [11]. We observed similarities in spirochete densities between animals in which cohorts of ticks were allowed to engorge or were interrupted during the bloodmeal, indicating the infectious dose was delivered during initial attachment. It was also unlikely that an interrupted bloodmeal resulted in the mouthparts and salivary gland components remaining at the bite site, causing an erroneous inoculum. Unlike ixodid ticks, *Ornithodoros* spp. fail to produce attachment cement [11], [29], and are relatively easy to remove. The cohort of ticks used in this study was also subsequently fed, indicating their mouthparts remained intact.

An interrupted bloodmeal may naturally occur, and salivary gland colonization is essential for *B. turicatae* transmission. Full engorgement by *O. turicata* was reported to be partially dependent on the attachment site and host activity [11], and both vector and likely mammalian hosts are active nocturnal feeders [25]–[27]. The transmission kinetics of *B. turicatae* suggested that spirochetes were localized within regions of salivary glands that would promote rapid transmission, for example, excretory ducts and the lumen of saliva producing acini [29]. Visualization of spirochetes by SEM indicated that acini lumen was a colonization site for *B. turicatae*, and while spirochetes may also localize in excretory ducts we did not visualize the bacteria within the ducts. Colonization of the acini lumen enables transmission through the saliva and increases the likelihood of continuing the spirochetes life-cycle when feeding on an active host.

Our findings and studies in the *B. hermsii-O. hermsi* model of relapsing fever spirochetes indicate that transmission and dissemination of the bacteria within the tick and mammal is unique [30]. For example, *B. burgdorferi*, the causative agent of Lyme borreliosis, initially colonizes the midgut of *Ixodes* spp. During the subsequent bloodmeal, spirochetes replicate in the midgut, migrate to the salivary glands, and transmit to the host, which takes approximately 48 hours [9], [31]. Upon entering mice, *B. burgdorferi* colonizes the attachment site for 48 hours prior to disseminating, as demonstrated by removing the bite site to prevent infection [32]. As *B. turicatae* enters the midgut, the spirochetes colonize the salivary glands during the following weeks given that *O. turicata* can subsequently transmit bacteria upon molting. Our findings suggest that the model of vector transmission and early mammalian infection of *B. turicatae* includes persistent colonization of salivary gland acini, and during the bloodmeal the bacteria are deposited at the bite site shortly after attachment. Within the host, *B. turicatae* disseminates into the blood during the time required for tick engorgement.

The ability of *B. turicatae* to remain infectious in a vector that can endure five years between feedings [21] indicates the importance of understanding the molecular mechanisms utilized by the spirochetes within the salivary glands and during the bloodmeal. Tick-transmitted bacteria adapt genetically when transiting between vector and mammal, with *B. burgdorferi* being the most characterized of these pathogens [2], [33],[34]. Studies have demonstrated the interplay between *B. burgdorferi* surface proteins with vector produced proteins. The tick receptor for OspA (TROSPA) is produced in the midgut and enables *B. burgdorferi* adhesion and colonization of the tissue [35]. *B. burgdorferi* also induces the up-regulation of tick genes including *salp15*, which encodes a 15 kDa salivary gland protein [36]. Salp15 acts as an immune suppressor and shields *B. burgdorferi* from antibody-mediated killing [36], [37]. Less is known regarding the mechanisms of vector adaptation and transmission by relapsing fever spirochetes. Mans and colleagues conducted a comparative study of salivary gland proteins between hard and soft ticks, reporting that the major protein families were conserved between Ixodidae and Argasidae [38]. However, little is known regarding the interplay between relapsing fever spirochetes and soft tick salivary proteins, and whether the saliva aids in bacterial infection of the mammal.

While the molecular mechanisms involved with relapsing fever spirochete transmission are still poorly understood, this study suggests that upon salivary gland colonization, *B. turicatae* is preadapted to infect mammalian blood. Future studies will focus on identification of gene products expressed by both ticks and spirochetes that enable salivary gland colonization and host transmission. Additionally, the bacterial dose delivered during the bloodmeal and the role of *O. turicata* saliva in enabling spirochete infection of the mammal will be determined. Collectively, these studies will further enhance our understanding of the mechanisms occurring during the tick-mammalian infectious cycle.

## Supporting Information

Figure S1 **Removal of the bite site.** After tick detachment (A) the bite site was removed using a 2 mm tissue punch (B and C).
(TIF)
