# Supplementary figures and images for: Transmission Dynamics of Borrelia turicatae from the Arthropod Vector
Source: PLoS Negl Trop Dis. 2014 Apr 3;8(4):e2767. doi: 10.1371/journal.pntd.0002767 (PMC3974661; doi:10.1371/journal.pntd.0002767)

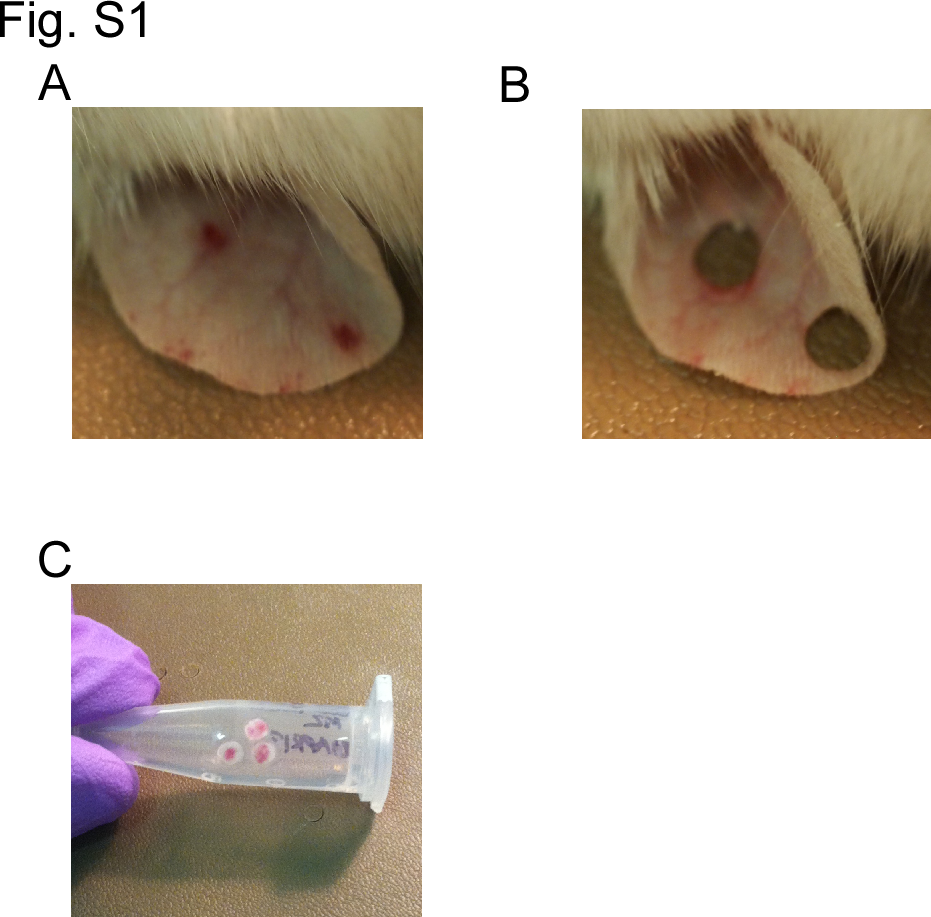

Supplement: Figure S1 — Removal of the bite site. After tick detachment (A) the bite site was removed using a 2 mm tissue punch (B and C). (TIF) [file pntd.0002767.s001.tif]
